# Biodegradation and metabolic pathway of fenvalerate by *Citrobacter freundii* CD-9

**DOI:** 10.1186/s13568-020-01128-x

**Published:** 2020-10-30

**Authors:** Jie Tang, Dan Lei, Min Wu, Qiong Hu, Qing Zhang

**Affiliations:** Key Laboratory of Food Biotechnology, School of Food and Biotechnology, Xihua University, Chengdu, 610039 Sichuan PR China

**Keywords:** Fenvalerate, *Citrobacter freundii* CD-9, Response surface methodology, Biodegradation metabolites, Metabolic pathway

## Abstract

Fenvalerate is a pyrethroid insecticide with rapid action, strong targeting, broad spectrum, and high efficiency. However, continued use of fenvalerate has resulted in its widespread presence as a pollutant in surface streams and soils, causing serious environmental pollution. Pesticide residues in the soil are closely related to food safety, yet little is known regarding the kinetics and metabolic behaviors of fenvalerate. In this study, a fenvalerate-degrading microbial strain, CD-9, isolated from factory sludge, was identified as *Citrobacter freundii* based on morphological, physio-biochemical, and 16S rRNA sequence analysis. Response surface methodology analysis showed that the optimum conditions for fenvalerate degradation by CD-9 were pH 6.3, substrate concentration 77 mg/L, and inoculum amount 6% (v/v). Under these conditions, approximately 88% of fenvalerate present was degraded within 72 h of culture. Based on high-performance liquid chromatography and gas chromatography-mass spectrometry analysis, ten metabolites were confirmed after the degradation of fenvalerate by strain CD-9. Among them, *o*-phthalaldehyde is a new metabolite for fenvalerate degradation. Based on the identified metabolites, a possible degradation pathway of fenvalerate by *C. freundii* CD-9 was proposed. Furthermore, the enzyme localization method was used to study CD-9 bacteria and determine that its degrading enzyme is an intracellular enzyme. The degradation rate of fenvalerate by a crude enzyme solution for over 30 min was 73.87%. These results showed that strain CD-9 may be a suitable organism to eliminate environmental pollution by pyrethroid insecticides and provide a future reference for the preparation of microbial degradation agents and environmental remediation.

## Introduction

Pyrethroids represent the second largest category of pesticides other than organophosphorus agents, accounting for approximately 20% of the global pesticide market. Pyrethroid insecticides are extracted from chrysanthemum plants and are a successful example of chemical pesticides developed from plant Giri et al. 2002; Laffin et al. 2010; Tripathi et al. 2004). Over the past 20 years, synthetic pyrethroid-based insecticides have been widely used in agricultural and non-agricultural settings in China (Saikia and Gopal 2004; Zhang et al. 2010). Fenvalerate represents the earliest-used pyrethroid insecticide in Chinese agriculture (Giri et al. 2002; Tripathi et al. 2004), and is widely used to control various pests owing to its high efficiency, low cost, and good light-exposure stability. However, with their long-term widespread application, pyrethroid pesticides are easily adsorbed by soil particles and transported by surface runoff, leaching, groundwater, and wind. This has resulted in agricultural non-point source pollution (Norum et al. 2010; Zhang et al. 2010) with high amounts of residues in agricultural products and foodstuffs, thus, posing a threat to human health. Pyrethroid insecticidal toxicity and residual toxicity are high. During the processing of agricultural and associated products, the accumulation of residues has a particularly significant effect on the living environment. Although pyrethroid pesticides are relatively non-toxic to mammals, recent studies have shown that they have accumulated toxicity, reproductive toxicity, and neurotoxicity. Long-term exposure in humans also carries the risk of carcinogenic, teratogenic, and mutagenic consequences (Laffin et al. 2010). Ahmad et al. (2009) found that excessive use of pyrethroid insecticides can cause DNA damage and increase the number of sperm head deformities in dwarf goats. Consequently, pyrethroid pesticides are neurotoxic substances, and their residues are harmful to the environment and human health (Soderlund et al. 2002; Tang et al. 2018b; Huang et al. 2019). Therefore, strategies to effectively eliminate pyrethroids to reduce environmental damage are worth pursuing.

Microbial degradation has been considered as a rapid, non-secondary polluting, and low-cost technology and is widely used to reduce pesticide pollution in the environment (Hu et al. 2019; Huang et al. 2020). Microbial degradation is mainly achieved by screening and isolating highly efficient degrading strains from pesticide-contaminated soil (Zhan et al. 2020). To date, different pyrethroid-degrading strains have been isolated by enrichment, direct isolation, and other technologies; these include *Bacillus* sp., *Alcaligenes* sp., *Micrococcus* sp., *Sphingomonas* sp., *Achromobacter* sp., *Aspergillus* sp. and *Pseudomonas* sp., etc. (Chen et al. 2011a; Guo et al. 2009; Liang et al. 2005; Maloney et al. 1993; Tallur et al. 2008; Tang et al. 2015). However, bioremediation efficiency and metabolic pathways involving native bacteria and fenvalerate have not been extensively explored, and are only rarely reported. Furthermore, studies concerning the biodegradation of fenvalerate (Cycon et al. 2016; Gill et al. 2015) and its degradation pathways are still rare.

*Citrobacter freundii* is widely distributed in nature and part of the normal flora in the intestines of humans and animals (mammals, birds, reptiles, and amphibians). In the current study, *C. freundii* has been shown to remove nitrogen and degrade naphthalene, phenanthrene, and ammonia in wastewater, adsorb heavy metals, and degrade pesticides such as parathion, chlorpyrifos, and *p*-nitrothiophenol (Pino et al. 2011a, b). To our knowledge, this is the first study regarding the biodegradation of pyrethroids by *C. freundii*.

The essence of microbial degradation of pyrethroids is the enzymatic reaction (Bhatt et al. 2020a). The degradation of pesticides by enzymes not only has the advantages of high speed, high efficiency, and good degradation effect on low concentration pollutants but also good tolerance by the environment (Tang et al. 2017). More importantly, the catalytic enzyme is a natural protein with no toxicity or side effects. It can be used to detoxify and remove pesticide residues in agricultural products (Bhatt et al. 2019). Moreover, research concerning degradation enzymes involving pyrethroid pesticides can not only further determine the degradation pathways and metabolic mechanisms of pyrethroid strains involved but also lay the foundations for future research on cloning, expression, and purification of such degradation enzymes.

The objectives of this study were (I) to isolate and characterize the fenvalerate degrading bacterial strain CD-9 from sludge by using enrichment culture, (II) to use a Box–Behnken experimental design to identify the optimal culture conditions for degradation by response surface methodology (RSM), (III) to elucidate the biodegradation mechanisms of fenvalerate by strain CD-9 and propose a possible metabolic pathway, and (IV) to extract and locate pyrethroid pesticide degrading enzymes.

## Materials and methods

### Chemicals and media

Fenvalerate (purity 96%) was obtained from Rongcheng Chemicals (Nanjing, China). Chromatographic grade acetonitrile was purchased from Ada-mas-Beta Reagent Co. (Shanghai, China). Dibutyl phthalate and 3-phenoxybenzoic acid (3-PBA) standard products (purity 98%) were obtained from TCI Reagent Factory (Shanghai, China). Other reagents used in this study, including *o*-phthalaldehyde, 4-chlorophenylacetic acid, and phenol, were purchased from Kelong Chemical Reagent Factory (Chengdu, China). All other analytical grade chemicals and solvents were purchased from Kelong Chemical Co. Standard solutions of pyrethroids and 3-PBA were prepared by dissolving in acetonitrile (10 g/L) for HPLC calibration.

Luria–Bertani (LB) medium contained 10.0 g of tryptone, 5.0 g of yeast extract, and 10.0 g of NaCl per liter of water. Mineral salt medium (MSM) contained 2.0 g of (NH_4_)_2_SO_4_, 0.2 g of MgSO_4_·7H_2_O, 0.01 g of CaCl_2_·2H_2_O, 0.001 g of FeSO_4_·7H_2_O, 1.5 g of Na_2_PO_4_·12H_2_O, and 1.5 g of KH_2_PO_4_ per liter of water were used in this study. The pH of both culture media was adjusted to 7.0.Enrichment medium was prepared as described by Liu et al. (2014). Liquid and solid LB medium and MSM were prepared according to Wang et al. and Teng et al. (2016, 2017). Tween 80 (0.2%, w/v) was added as an emulsifier before sterilization at 121 °C for 20 min. All solutions were sterilized by filtration (0.45 µm membrane) and diluted to the desired concentration in culture medium (Chen et al. 2011b).

### Enrichment, isolation, and screening of fenvalerate degrading strains

An enrichment culture technique was used to isolate fenvalerate degrading strains. One gram of sludge sample obtained from Sichuan Pesticide Chemical Co. Ltd. was transferred to a 250 mL Erlenmeyer flask containing 50 mL of sterilized enrichment medium and 25 mg/L of fenvalerate. The enrichment culture flask was incubated in an incubator with rotary agitation at 180 rpm and 30 °C for 5 days. After 5 days, 5 mL of enrichment culture was transferred to new Erlenmeyer flasks containing 50 mL of fresh MSM and 50–2000 mg/L fenvalerate. After eight rounds of transfer, the enrichment medium was serially diluted, plated, and incubated on MSM plates containing 100 mg/L fenvalerate, and finally a single colony was purified three times on LB agar media (Tang et al. 2018a). An equal volume of acetonitrile was added to 1 mL of degradation system and transferred to a 10 mL tube. Ultrasound (40 kHz, 100 W) assisted extraction for 30 min. After centrifuging the mixture at 12,000 rpm for 10 minutes, the supernatant was collected and filtered through a 0.45 µm organic phase membrane filter. Finally, samples were analyzed by HPLC (Waters 2690, United States). The fenvalerate degradation rate was calculated according to the following equation (Eq. 1), and one of the effective strains, termed CD-9, was selected for further studies.

1 $${\text{Degradation rate }}\left( \% \right){\text{ }} = {\text{ }}\left( {{\text{1 }} - C/C_{0} } \right){\text{ }} \times {\text{ 1}}00\%$$

where *C* is the fenvalerate content of the inoculation medium, and *C*_*0*_ is the fenvalerate content of the control medium.

### Identification of fenvalerate-degrading strains

#### Morphological identification and physiological and biochemical tests of strains

The colony formation characteristics, including size, color, surface, and edge, of strain CD-9 were identified using an optical microscope (Olympus Japan). To further understand such morphology, cells were examined using a scanning electron microscope (Japan Electronics Co., Ltd., Japan). Physio-biochemical assays were tested with reference to *Bergey’s Handbook of Assay Bacteriology* (Holt et al. 1994).

#### Molecular biological identification of strain CD-9

Sequencing of 16S rRNA, the most conserved region of DNA in prokaryotes, was used to identify strain CD-9 (Festa et al. 2013). Total genomic DNA was extracted using the TIANamp Bacterial DNA Kit (Tiangen Biotech Co., Beijing, China). PCR was used to amplify the 16S rRNA gene using the universal forward and reverse primers Eu27F (5′-AGAGTTTGATCCTGGCTCAG-3′) and 1490R (5′-GGTTACCTTGTTACGACTT-3′), respectively. A Gel Extraction kit was used to recover the amplified 16S rRNA. After quantification, the PCR products were sent to Tsingke Biological Technology Co. (Chengdu, China) for DNA sequencing. NCBI BLAST (http://www.ncbi.nlm.nih.gov/blast/) was used to compare 16S rRNA sequences. A phylogenetic tree was constructed using MEGA 7.0 software and the neighbor-joining method, with 1000 repeated bootstrap analyses.

#### Growth and degradation curve of strains

*Citrobacter freundii* strain CD-9 was stored in 20% glycerol at −50 °C. Before each experiment, bacterial strains were thawed and grown in 250 mL Erlenmeyer flasks containing 50 mL of sterile LB medium. The culture was then incubated in a 30 °C rotary shaker at 180 rpm for 24 h. Bacterial cells at the later stage of exponential growth were collected by centrifugation (10 min, 10,000 rpm) at 4 °C. The cell precipitate was washed three times with 0.9% sterile saline, resuspended in sterile water, and then adjusted to an optical density (OD) value at 600 nm of approximately 1.0 to prepare a liquid inoculums (Tang et al. 2018a).

Next, 5 mL of bacterial solution was added to 95 mL MSM medium, utilizing 50 mg/L of fenvalerate as the sole carbon source. An equivalent volume of sterile saline was used as a blank control. The culture was shaken at 30 °C and 180 rpm for 108 h. By measuring the OD_600_ using an UV-spectrophotometer, the growth of strain CD-9 was checked every 2 h for the first 12 h, and tested every 12 h for the next 96 h. The concentration of residual fenvalerate was determined by HPLC.

### Optimization of conditions for fenvalerate degradation by CD-9

The RSM designed by Box–Behnken, was used to optimize the key factors and interactions affecting CD-9 degradation of fenvalerate. To obtain optimal conditions for fenvalerate degradation, based on the results of preliminary one-factor-at-a-time experiments, the initial pH, substrate concentration, and inoculation amount were selected as independent variables (Chen et al. 2013). The following conditions: the initial pH 5.5–7.5, fenvalerate concentration 65 mg/L–85 mg/L and inoculum amount 5%–7%. After 72 h of incubation, degradation rates were determined by HPLC. A three-variable Box–Behnken design was used for this experiment, which had three replicates at the center point to estimate the experimental error, and noninoculated cultures served as controls. The experimental data was analyzed according to Design–Expert software (version 10.0 Stat-Ease Inc., Minneapolis, Minnesota, USA) to construct a secondary model. The initial substrate pesticide concentration was 100 mg/L.

### Metabolites of fenvalerate degradation by CD-9

The CD-9 strain was transferred to LB medium containing 75 mg/L fenvalerate with an inoculum size of 5% (v/v), and incubated at 30 °C, 180 rpm on a rotary shaker. After 72 h of incubation, 20 mL of the sample was centrifuged at 10,000 rpm for 10 min and extracted after acidification to pH 2 with 2 M HCl, according to Tallur et al. (2008). Then, an equal volume of ethyl acetate was added, and extraction was assisted by ultrasound (40 kHz, 100 W) for 30 min, and left to stand at room temperature for 10 min. The upper organic phase was collected and dehydrated with anhydrous sodium sulfate, then evaporated to dryness on a rotary evaporator, and finally redissolved in 2 mL of methanol (Tallur et al. 2008). Fenvalerate metabolites were analyzed by gas chromatography-mass spectrometry (GC-MS) (Bhatt et al. 2016) based on retention times (RTs) and peak areas of pure standards (Yu et al. 2013).

### Analytical methods

#### HPLC conditions and analysis

LC-20AT was used to determine the fenvalerate content. An HPLC instrument (Shimadzu, Kyoto, Japan) was equipped with an LC-20AT pump, a ZORBAX eclipse plus C18 column (4.6 mm × 150 mm, 5 µm), and an SPD-M20A detector (Tang et al. 2018a). A series of standard mixture solutions with a concentration of 1.0 to 50.0 mg/L was prepared by accurately diluting a standard solution of fenvalerate (10 g/L) in acetonitrile. The mobile phase consisted of solvent A (0.01% ammonium acetate (v/v) in distilled water) and solvent B (acetonitrile). After pretreatment of the samples as described above, 10 µL of each sample was injected into the HPLC system. The solvent flow rate was set at 0.5 mL/min. The gradient of the mobile phase was maintained using 85% solvent B for 7 min, linearly increased to 90% solvent B in 0.5 min, held for 7.5 min, then returned to 85% solvent B in 5 min, and finally stabilized at 21 min. The detection wavelength was 210 nm, and the RT of fenvalerate was 8.689 min. Linear regression was performed on the corresponding peak area (Y) by plotting fenvalerate concentration (X, mg/L), and the following calibration curve equation was obtained: Y = 38,596X − 81,371, R^2^ = 0.999. The precision was evaluated by analyzing the relative standard deviation (RSD) in five tests (Wang et al. 2019; Chen et al. 2016). The mean recoveries for the tested compounds were found to be in the range of 96.58–99.08% with RSDs of 0.91–1.65%, indicating that this method has good precision.

#### GC-MS conditions and analysis

Fenvalerate metabolites were identified using a Shimadzu GC2010 Plus gas chromatograph coupled to a Shimadzu MS2010 Plus mass spectrometer in electron ionization mode (70 eV) with a DB-5 column (30.0 m × 0.25 mm × 0.25 mm). The samples (extracts of fenvalerate, phenol, and catechol) were filtered through a 0.45 µm organic phase membrane filter and analyzed on an Agilent 6890N/5975 GC-MS system. Helium (99.999%) was used as GC carrier gas at a constant flow rate of 1.5 mL/min. The injection volume was l µL. Injection mode was splitless at 250 °C. The temperature of the transmission line and the ion source are 250 °C and 280 °C, respectively. GC oven was programed with the initial temperature of 60 °C for 2 min, followed increase to 190 °C at 8 °C min^− 1^ ramp, holding for 1 min, then increased to 230 °C at 10 °C min^− 1^ ramp, holding for 4 min, and finally increased to 270 °C at 10 °C min^− 1^ ramp, holding for 25 min. Metabolites identified by GC-MS analysis were matched to real-world standard compounds from the National Institute of Standards and Technology (NIST, USA) library database (Chen et al. 2013).

### Preparation of the CD-9 strain crude enzyme solution

Cryogenically preserved strain CD-9 was cultured in LB liquid medium for 24 h for activation (Tang et al. 2015). The activated CD-9 strain was inoculated into an enrichment medium that contained 100 mg/L of fenvalerate, cultured at 30 °C, placed in a 180 rpm shaker for 2 days, and then centrifuged at 10,000 rpm for 10 min. The supernatant and slime were collected separately to further detect the activity of intracellular and extracellular enzymes according to the research of Bhatt et al. (2020a). The supernatant was incubated at 4 °C overnight, and ammonium sulfate was added until 100% saturation. Then, the precipitate was collected by centrifugation at 10,000 rpm for 10 min (Zhang et al. 2019). After dissolving in a small volume of 0.02 mol/L phosphate buffered saline (PBS), the precipitate was dialyzed against the same buffer without SO_4_^2−^ and concentrated with PEG20000 to obtain a crude extracellular enzyme solution (Yehia et al. 2015). The collected sludge was washed with 0.02 mol/L PBS (pH 7.0) (1: 3, v/v), placed on ice, and homogenized using a YIY-UL500W-Lulrasonic homogenizer (Zhang et al. 2019). The homogenized bacteria were crushed and processed for six cycles. Each cycle consisted 15 s processing followed by a 5 s rest (Zhang et al. 2019). The solution of ruptured bacteria was centrifuged at 10,000 rpm for 10 min at 4 °C. The resulting supernatant was a crude intracellular enzyme solution, and the precipitate was resuspended in PBS (1: 3, v/v), forming the crude enzyme solution derived from intracellular debris.

### Localization of pyrethroid degrading enzymes

The pyrethroid degrading enzyme was localized using pyrethroid pesticides as the substrate. A 50 mL of culture medium, was used to extract the extracellular crude enzyme solution, intracellular crude enzyme preparation, and cell debris crude enzyme solution, according to the method described above.

The location of pyrethroid degradation enzymes was determined according to the methods of Maloney et al. (1993). The reaction system comprised 3.0 mL, including 2.8 mL of 20 mmol/L Tris-HCl buffer solution and 0.1 mL of pyrethroid pesticide (0.6 mg/mL), at pH 8.0. The mixture was placed in a constant temperature water bath preheated at 30 °C for 10 min. Then, 0.1 mL of crude enzyme solution was added and shaken in a water bath at 30 °C for 30 min. The enzyme reaction was stopped by adding 0.1 mL of 1.0 mol/L HCl solution. Three replicates were set up for each treatment, and add the HCl solution directly to the crude enzyme without incubation as the control. Finally, samples were processed according to the above pretreatment methods, and residual pesticide was detected by HPLC, after which calculated the pesticide degradation rate using Eq. 1.

### Statistical analysis

Statistical analysis was performed using Origin software (version 8.5). Statistical significance was determined by one-way analysis of variance (ANOVA) test at *p* < 0.05 to examine specific differences between treatments. ChemDraw Professional software (version 16.0) was used to graphically depict the fenvalerate degradation pathway. Each experiment was repeated three times, with no inoculation conditions as the control, and the results are expressed as the mean of three replicates ± relative standard deviation.

## Results

### Isolation and identification of fenvalerate degrading bacteria

Taking fenvalerate as the research object, five strains of bacteria that are able to effectively degrade pyrethroid pesticides were obtained by enrichment, separation and purification from sludge samples. *C. freundii* CD-9 (Collection number: CGMCC 20,106, GenBank accession number: MN629225.1), *Pseudomonas aeruginosa* CD-13, *S. acidophilus oligotrophus* HF-17-1, *Alcaligenes* HF-15, and *Maltophilia* HF-1-2-1. The strain CD-9 exhibited a high degradation rate of fenvalerate, and was able to degrade 76.37% fenvalerate (100 mg/L) within 96 h, which has not been mentioned in previous studies.

**Fig. 1:**
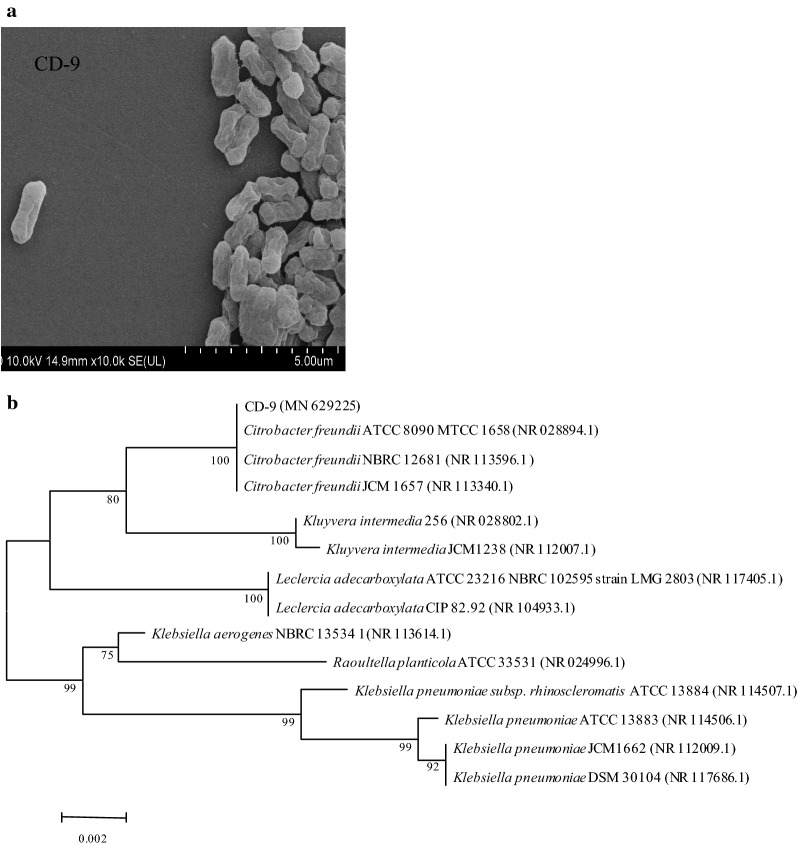
**a** Morphology of *Citrobacter freundii* cells by scanning electron microscopy. **b** Phylogenetic analysis of *C. freundii* CD-9 based on the 16S rRNA sequence. The numbers shown in parentheses are GenBank sequence accession numbers. The numbers at the nodes indicate bootstrap values from neighbor-joining analysis of 1000 resampled data sets. The bar represents sequence divergence

The morphology of strain CD-9 was observed by scanning electron microscopy (Fig. 1a). The CD-9 strain is a short and thick *Bacillus* sp., with dimensions of 2–4 µm in length and 1–1.5 µm in width. Cells can exist alone or can be arranged in pairs or short chains without spores. Colonies grown on LB agar plates for 24 h were beige, round, sticky convex and smooth with entire margins. The results of physiological and biochemical tests showed that the CD-9 strain was a gram-negative bacterium. It was positive in tests or reactions such as nitrate reducibility, urea hydrolysis, and catalase, methyl red, hydrogen sulfide production, and lactose and sucrose fermentation, whereas it was negative for oxidase activity, indole reaction, VP test, lysine decarboxylation, gelatin liquefaction, starch hydrolysis, arginine hydrolysis and ornithine hydrolysis (Table 1). Phylogenetic analysis of the 16S rRNA gene sequence using BLAST (Fig. 1b) showed that strain CD-9 was associated with *C. freundii* 8090MTCC1658 (NR028894.1), and *C. freundii* NBRC12681 (NR113596.1) shared 99% homology. Thus, CD-9 was identified as *C. freundii* (MN629225).

**Table 1:** Characteristics of physiological and biochemical assays of strain CD-9

Characteristic	Result	Characteristic	Result
Liquid culture	Turbid liquid	Gram staining	Negative
Methyl red	+	Oxidase	−
Sportiness	+	Nitrate	+
Catalase	+	Indole	−
Hydrogen sulfide	+	VP test	−
Gelatin liquefaction	−	Esculin hydrolysis	−
Ornithine decarboxylase	−	Lysine decarboxylase	−

+, tested positive/utilized as substrate; −, tested negative/unutilized as substrate

### Utilization of fenvalerate as sole carbon source for growth of CD-9

**Fig. 2:**
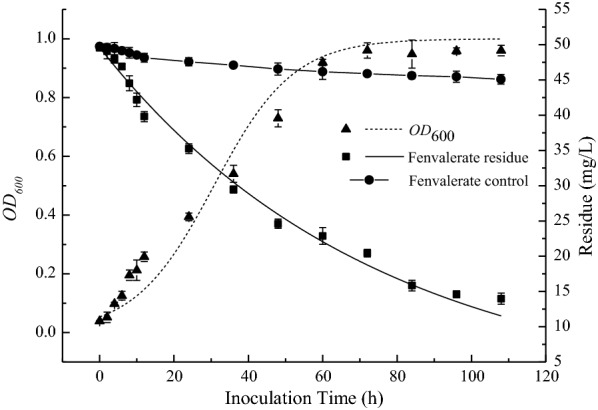
Growth and fenvalerate degradation of *C. freundii* CD-9

Figure 2 illustrates how the growth and degradation of stain CD-9 were investigated in MSM containing 50 mg/L fenvalerate as the sole carbon and energy source. The results showed that the degradation of fenvalerate was related to the growth of *C. freundii* CD-9. Approximately 9.0% degradation was observed in the non-inoculated control. The strain experienced lag, exponential, and plateau growth phases, and > 75% of fenvalerate present was degraded within 96 h. A cell growth kinetic model (Eq. 2) and a first-order degradation kinetic model (Eq. 3) were fitted to the experimental values to obtain the following:

2 $${X_{CD - 9}}~ = 0.05314{e^{0.0937t}}/\left( {1{-}0.05534\left( {1{-}0.05314{e^{0.0937t}}} \right)} \right),{\mu _m} = 0.0937{h^{{-}1}},\;{X_0} = 0.05314\;and\;{X_m} = 0.9602;\;{C_{CD - 9}} = 49.60011{e^{{-}0.01351t}},\;k = 0.01351,\;\;{t_{1/2}} = 51.33h,\;{R^2} = 0.9836. X = {X_0}{e}^{u_m }{^t}/[1 - \left( {{X_0}/{X_m}} \right)(1 - {e}^{u_m }{^t})]$$

where *X* is the predicted value of fenvalerate degradation by *C. freudii* CD-9 (%), *X*_*0*_ is the initial cell concentration (OD_600_), *X*_*m*_ is the maximum cell concentration (OD_600_), *t* is the culture time (h), and µ_*m*_ represents the maximum specific growth rate (h^− 1^).

3 $${C_t} = {C_0} \times {\rm{ }}{e^{ - kt}}\quad {t_{1/2}} = \ln 2/k$$

where *C*_*0*_ represents the initial fenvalerate concentration (mg/L), *C*_*t*_ represents the residual fenvalerate concentration (mg/L) at time t, *t* represents the degradation time (h), *k* indicates the degradation rate constant (h^− 1^), and *t*_*1/2*_ represents the half-life of fenvalerate.

### Optimization of fenvalerate degradation conditions

Degradation of fenvalerate depends on a number of factors, such as culture conditions, inoculation volume and presence of other compounds (Chen et al. 2013; Wan et al. 2010). Based on the preliminary results of the single factor experiment, the RSM experiment included three factors, pH, substrate concentration, and inoculum amount (v/v). The three parameters were taken at a central coded value considered as zero and investigated at three different levels (−1, 0, and + 1). The obtained fenvalerate degradation data represented the effects of the combination of these three variables at different levels. Table 2 lists experimental design variables corresponding to the response of fenvalerate residues. Subsequently, the data were analyzed using a response surface-regression procedure. The highest degradation of 89.6% and the lowest of 56.9% were observed. A quadratic polynomial function was fitted to the experimental values to obtain the following equation (Eq. 4):

4 $${\text{Fenvalerate degradation}}\;\left( \% \right)\; = - \;1419.13125 + 69.82\;{\text{pH}}\; + 18.9938\;{\text{fenvaerate}}\;{\text{concentration}}\; + 194.43\;{\text{inoculum}}\;{\text{amount}} + 0.2625\;{\text{pH}}\; \times {\text{fenvalerate}}\;{\text{concentration}}\; - 1.55\;{\text{pH}} \times {\text{inoculum}}\;{\text{amount}} - 0.5925\;{\text{fenvalerate}}\;{\text{concentration}} \times {\text{inoculum}}\;{\text{amount}} - 6.69\;{\text{p}}{{\text{H}}^2}\; - 0.1117\;{\text{fenvalerate}}\;{\text{concentratio}}{{\text{n}}^2} - 11.89\;{\text{inoculum}}\;{\text{amoun}}{{\rm{t}}^2}$$

**Table 2:** Optimization of fenvalerate degradation using response surface methodology

Run	pH	Fenvalerate concentration	Inoculum amount	Fenvalerate degradation (%)
1	0	0	0	85.2
2	−1	1	0	76.1
3	0	0	0	89.4
4	0	−1	−1	58.1
5	0	0	0	89.6
6	1	−1	0	58.9
7	0	1	1	59.9
8	0	−1	1	63.4
9	−1	0	1	77.4
10	1	1	0	71.7
11	0	1	−1	78.3
12	−1	0	−1	78.8
13	0	0.	0	87.8
14	−1	−1	0	73.8
15	0	0	0	87.9
16	1	0	1	56.9
17	1	0	−1	64.5

pH: −1(5.5), 0 (6.5), 1 (7.5); Fenvalerate concentration (mg/L): −1 (65), 0 (75), 1 (85); Inoculum amount (%): −1 (5.0), 0(6.0), 1 (7.0)

Analysis of variance (ANOVA) is the key to testing the importance and sufficiency of the model (Ghevariya et al. 2011). Table 3 shows our ANOVA results fitted to a quadratic polynomial model. The determination of a correlation coefficient of R^2^ = 0.9799 indicated that approximately 97.99% of responses were covered by the model, demonstrating that the model was able to predict values that were consistent with the experimental values. A very low probability value (*P* < 0.05) and a very high F value (*F* = 37.83) indicated that the model parameters were significant in predicting the response variable. Square terms of pH, fenvalerate concentration, and inoculum amount and interaction terms of fenvalerate concentration × inoculum amount showed significant effects (*P* < 0.05) on fenvalerate degradation by strain CD-9, however, pH × fenvalerate concentration and pH × inoculum amount played insignificant role (*P* > 0.05) in degradation.

**Table 3:** Analysis of variance (ANOVA) for the fitted quadratic model for fenvalerate biodegradation

Source	Sum of squares	Degrees of freedom	Mean square	*F*- Value	*P*-Value
Model	2182.22	9	242.47	37.83	< 0.0001
A-pH	365.85	1	365.85	57.09	0.0001
B- Fenvalerate concentration	126.41	1	126.41	19.72	0.0030
C-Inoculum amount	61.05	1	61.05	9.53	0.0177
A × B	27.56	1	27.56	4.30	0.0768
A × C	9.61	1	9.61	1.50	0.2603
B × C	140.42	1	140.42	21.91	0.0023
A^2^	188.45	1	188.45	29.41	0.0010
B^2^	524.87	1	524.87	81.90	< 0.0001
C^2^	595.25	1	595.25	92.88	< 0.0001
Residual	44.86	7	6.41		
Lack of fit	32.45	3	10.82	3.49	0.1294
Pure error	12.41	4	3.10		
Total	2227.08	16			

R^2^ = 0.9799; adjusted R^2^ = 0.9540; coefficient of variation = 3.42%; P-Value < 0.05 indicates that the model terms are significant

**Fig. 3:**
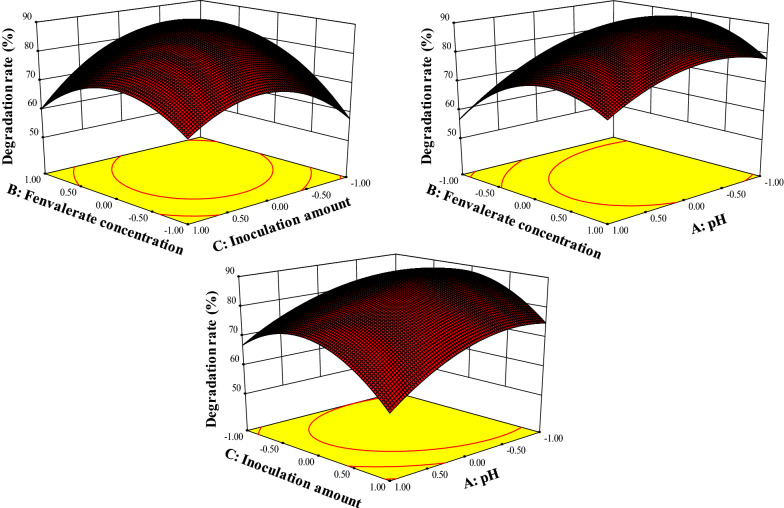
Three-dimensional plots of a quadratic model for fenvalerate degradation by strain CD-9

For a better understanding of the results, a 3D response surface plot is shown in Fig. 3, in which the theoretical maximum fenvalerate degradation rates were relatively similar for the three graphs. The center points in the contour plots represent the greatest potential for degradation of fenvalerate. Thus, the optimum culture conditions for fenvalerate degradation by strain CD-9 were concluded to be pH of 6.3, substrate concentration of 77 mg L^− 1^ and inoculum amount of 6%. In the optimized degradation system, the degradation rate of fenvalerate can reach 88%, which is basically consistent with the model prediction value (89.69%). In this study, a quadratic polynomial model (Eq. 4) was successfully established, and the degradation rate of strain CD-9 was improved with few experiments and the least resources.

### Identification of fenvalerate degradation metabolites

**Fig. 4:**
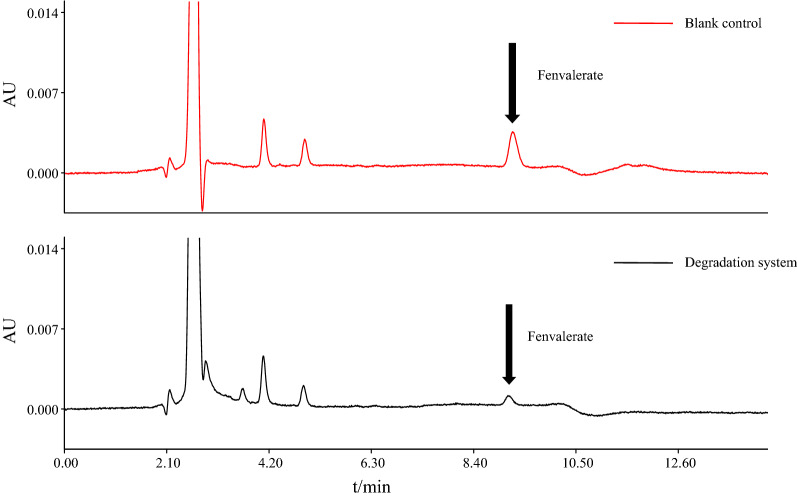
Comparison chart of before and after fenvalerate degradation (high-performance liquid chromatography [HPLC])

**Fig. 5:**
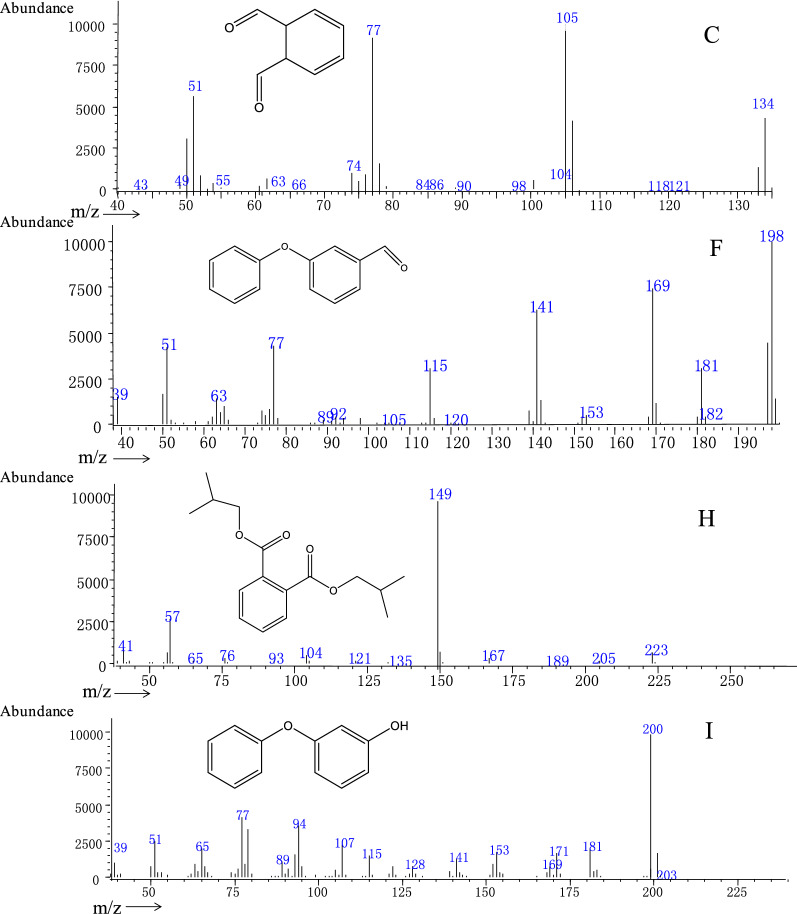
Gas chromatography-mass spectrometry (GC-MS) analysis of metabolites produced from fenvalerate degradation by strain CD-9. (C, F, H, I) Characteristic ions of compounds C, F, H, and I in GC-MS. Retention times of each compound were 14.732, 23.152, 25.408, and 28.825 min, respectively, and the compounds were identified as *o*-phthalaldehyde, 3-phenoxybenzaldehyde, dibutyl phthalate, and 3-phenoxybenzyl alcohol, respectively

To explore the mechanisms of fenvalerate degradation by *C. freundii* CD-9, the metabolites from fenvalerate degradation were extracted and confirmed by HPLC and GC-MS. After 48 h of incubation under optimal conditions, the HPLC results according to Fig. 4 showed that 76% of the pesticide substrate was degraded by CD-9. Through GC-MS detection, based on the similarity of their fragment RTs and molecular ions corresponding to those of authentic compounds in the NIST library database, four main degradation products were identified (Fig. 5). The RTs and quantifier ions of ten compounds that appeared during degradation are listed in Table 4. Compounds A-J were identified as phenol (A), phenylacetaldehyde (B), *o*-phthalaldehyde (C), *p*-hydroxyphenylacetic acid (D), 2-methylhexanoic acid (E), 3-phenoxybenzaldehyde (F), α-cyano-3-phenoxybenzyl alcohol (G), dibutyl phthalate (H), 3-phenoxybenzyl alcohol (I), and 3-chlorophenylacetic acid (J). Yang et al. (2018) reported that, during the degradation process, 3-benzylhydroxybenzyl alcohol is readily oxidized to form 3-PBA. Zhan et al. (2018) reported for the first time that compound H is present in the 3-PBA biodegradation pathway. However, this is the first study in which compound C was detected in the biodegradation pathway of fenvalerate. The similarity between compound J and the standard was only 62%, which may be because of the small chromatographic peak of compound J and background ion interference. The results showed that compound J was difficult to extract as it had a low content in the degradation mixture.

**Table 4:** Identification of intermediate metabolites of fenvalerate by gas chromatography-mass spectrometry (GC-MS)

Serial number	Retention time (min)	Similarity (%)	Chemical formula	Intermediate metabolites
A	9.725	74	C_6_H_6_O	Phenol
B	11.133	84	C_8_H_8_O	Phenylacetaldehyde
C	14.732	79	C_8_H_6_O_2_	*o*-Phthalaldehyde
D	18.658	88	C_8_H_8_O_3_	*p*-Hydroxyphenylacetic acid
E	19.975	84	C_7_H_14_O_2_	2-Methylhexanoic acid
F	23.152	90	C_13_H_10_O	3-Phenoxybenzaldehyde
G	23.208	85	C_14_H_11_NO_2_	α-Cyano-3-phenoxybenzyl alcohol
H	25.408	90	C_16_H_22_O_4_	Dibutyl phthalate
I	28.825	74	C_13_H_12_O_2_	3-Phenoxybenzyl alcohol
J	30.767	62	C_8_H_7_ClO_2_	3-Chlorophenylacetic acid

The intermediate metabolites dibutyl phthalate, 3-PBA, *o*-phthalaldehyde, and phenol were added as substrates to the MSM liquid medium, and *C. freundii* CD-9 was inoculated into the medium to optimize the culture conditions. After 48 h of incubation, the 3-PBA substrate was extracted and characterized by GC-MS. It was found that in the degradation system involving 3-PBA, dibutyl phthalate, 3-phenoxybenzaldehyde, and *o*-phthalaldehyde were detected (Table 5), which indicated that 3-PBA could be degraded by *C. freundii* CD-9. The peak areas of the remaining three intermediate products were measured by HPLC, and the differences in peak areas between the control and experimental groups were compared (Table 6). It was found that *C. freundii* CD-9 could effectively degrade dibutyl phthalate, *o*-phthalaldehyde and phenol. In summary, *C. freundii* CD-9 could be grown with dibutyl phthalate, 3-PBA, *o*-phthalaldehyde, or phenol as the sole carbon source and could continuously degrade intermediate metabolites, which indicated that the metabolites of fenvalerate are difficult to accumulate, thus, providing a new solution for complete degradation.

**Table 5:** Chromatographic characteristics of degradation products of 3-PBA generated by *Citrobacter freundii* CD-9

Serial number	Retention time (min)	Similarity (%)	Chemical formula	Chemical compound
a	14.732	79	C_8_H_6_O_2_	*o*-Phthalaldehyde
b	23.208	82	C_13_H_10_O	3-Phenoxybenzaldehyde
c	27.083	94	C_16_H_22_O_4_	Dibutyl phthalate

**Table 6:** High-performance liquid chromatography (HPLC) analysis of intermediate degradation products of fenvalerate generated by *Citrobacter freundii* CD-9

Metabolites	Control (mAU.s)	Experiment (mAU.s)
Phenol	1,895,118 ± 0.068	510,479 ± 0.049
*o*-Phthalaldehyde	100,248 ± 0.059	56,741 ± 0.007
Dibutyl phthalate	127,921 ± 0.024	33,191 ± 0.013

**Fig. 6:**
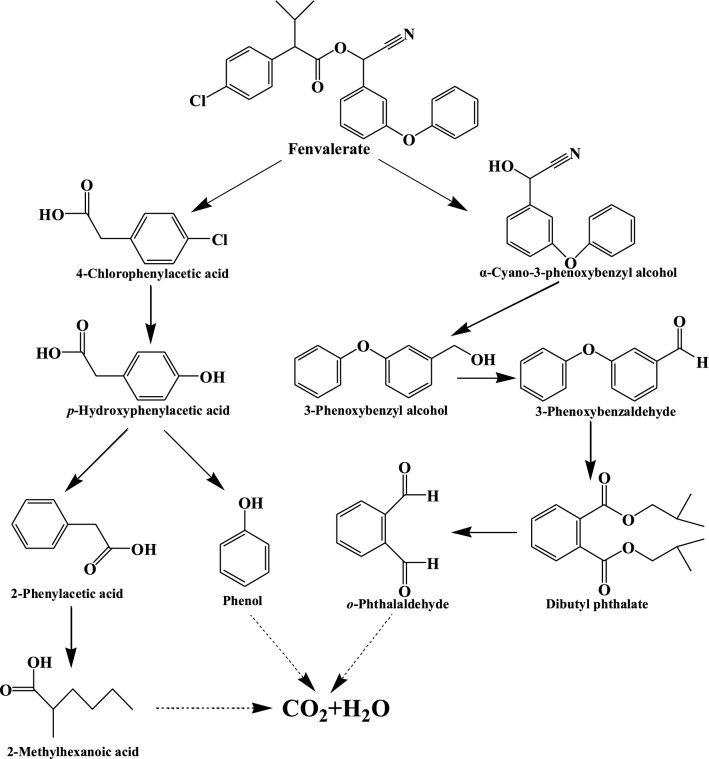
Proposed pathway for degradation of fenvalerate by strain CD-9

Based on analysis of the chemical structures of the metabolites formed during the degradation process, a possible pathway for the degradation of fenvalerate by *C. freundii* CD-9 was proposed (Fig. 6). In the proposed system, fenvalerate is firstly degraded to 3-chlorophenylacetic acid (J) and α-cyano-3-phenoxybenzyl alcohol (G) through ester linkage hydrolysis. This is commonly used by a variety of microorganisms to destroy the insecticidal activity of pyrethroids, leading to their detoxification (Lin et al. 2011). 3-Chlorophenylacetic acid is then converted to 4-hydroxyphenylacetic acid (D) through the hydrolysis of halogen elements and finally to phenol (A) and phenylacetaldehyde (B), followed by benzene ring cleavage to form 2-methylhexanoic acid (E). At the same time, because the structure of α-cyano-3-phenoxybenzyl alcohol (G) is unstable in the environment, it will quickly oxidize to form 3-phenoxybenzaldehyde (F), and then 3-phenoxybenzaldehyde is cleaved into *o*-phthalaldehyde (C), possibly by cleavage of the aromatic ring by dibutyl phthalate (H) (Zhan et al. 2018). It is worth noting that in the complete metabolic pathway that we deduced, in addition to the hydrolysis of the ester bond, *C. freundii* CD-9 further reduced the toxicity of pesticides through diaryl bond cleavage, thereby accelerating fenvalerate degradation. There were no persistent accumulative products during fenvalerate degradation.

### Cellular localization of pyrethroid degrading enzymes

Degradation characteristics of pyrethroids in the crude intracellular enzyme solution, crude cell debris solution, and extracellular crude enzyme solution of *C. freundii* CD-9 were measured, and the results are shown in Table 7. The degradation rates of the intracellular crude enzyme solution against fenvalerate (73.87%), beta-cypermethrin (67.90%), and bifenthrin (66.00%) exceeded 50%, whereas the extracellular enzyme was highly efficient on only bifenthrin (7.87%). The degradation rate of pyrethroid by the crude enzyme solution of cell debris was > 20%, which may be owing to certain degrading enzymes located on cell walls. Thus, the degradation of pyrethroid pesticides by the CD-9 strain was attributable to the intracellular enzyme.

**Table 7:** Degradation of pyrethroids by *Citrobacter freundii* CD-9 enzyme solution

Degradation rate (%)	Intracellular crude enzyme solution	Cell debris crude enzyme solution	Extracellular crude enzyme solution
Fenvalerate	73.87 ± 0.51	25.58 ± 0.90	1.11 ± 1.60
Bifenthrin	66.00 ± 1.04	20.23 ± 0.57	7.87 ± 1.36
Beta-cypermethrin	67.90 ± 0.76	23.57 ± 2.21	5.72 ± 0.88

## Discussion

Microbes play an important role in the degradation of pollutants (Hu et al. 2020). As far as we know, although there have been many studies on bacterial and fungal degradation of pyrethroid pesticides (Chen et al. 2012; Saikia and Gopal 2004; Tang et al. 2017; Zhan et al. 2018; Bhatt et al. 2020c), to our knowledge, there are no studies on the resistance and degradation of fenvalerate in *Citrobacter*. In this study, a CD-9 strain with high potential for fenvalerate degradation was isolated and screened. We observed the growth of this strain by using a light microscope and performed physiological and biochemical tests, in combination with 16S rRNA molecular characterization, and identified the CD-9 strain as *C. freundii* (Fig. 1).

RSM provides reliable information for optimizing key process parameters to enhance biodegradation by applying polynomial equations of empirical models to experimental data (Bezerra et al. 2008; Bhatt et al. 2020d). Previous studies have shown that pH, substrate concentration, and inoculum volume can affect the effective biodegradation of heterologous chemicals (Chen et al. 2011b, 2012). During this study, based on single factor results, the RSM based on a Box–Behnken design was successfully explored to improve the biodegradation process by strain CD-9, and optimum conditions for enhancing biodegradation were determined to be initial of pH of 6.3, fenvalerate concentration of 77 mg/L, and an inoculation amount of 6% (v/v). In this optimized degradation system, the degradation rate of fenvalerate could attain 88%. Similar enhanced degradation enabled through RSM has been reported for a variety of microbes such as *Bacillus subtilis* BSF01 (Xiao et al. 2015), *Brevibacillus parabrevis* BCP-09 (Tang et al. 2018a), and *Acinetobacter baumannii* ZH-14 (Zhan et al. 2018).

Metabolomics is considered to be a valuable tool for studying biodegradation and conversion pathways of pollutants (Wang et al. 2018). It is well established that hydrolysis plays an important role in the biodegradation of pyrethroid pesticides, which may be attributed to the breakage of its ester bonds (Stok et al. 2004; Tang et al. 2017; Zhang et al. 2011). In this study, fenvalerate was first hydrolyzed by cleaving ester bonds to produce the main intermediate products 3-phenoxybenzaldehyde and butyl 1,2-phthalate (Fig. 5). 3-Phenoxybenzaldehyde is a common by-product of pyrethroids (Yang et al. 2018) and is also detected during the biodegradation of fenvalerate (Chen et al. 2011b), deltamethrin (Chen et al. 2011c), cyhalothrin (Chen et al. 2015), and beta-cypermethrin (Tang et al. 2018a). However, only Yang et al. (2018) and Zhan et al. (2018) reported biodegradation of pyrethroids to obtain the metabolite butyl phthalate. The biodegradation of fenvalerate to obtain the metabolite *o*-phthalaldehyde has not been reported. It is worth noting that strain CD-9 has the ability to degrade intermediate products (Table 5), and no further metabolic products accumulated at the end of the experiment, indicating that the isolate may have a complete metabolic pathway for fenvalerate degradation and metabolism of esters. According to the metabolite analysis in this study, the degradation pathway involving fenvalerate in strain CD-9 has been proposed (Fig. 6), which may provide new insights into the biological cycle of pesticides on soil.

In the process of biodegradation, the microenvironment may directly damage the microorganisms present, thereby affecting the degradation, whereas the direct use of degrading enzymes can help eliminate such effects. Gouda et al. (2018) and García et al. (2017) showed that pesticide degradation enzymes are efficient, safe, environmentally resistant, and have a wide range of degradation capabilities. Tallur et al. (2008) and Bhatt et al. (2020b) determined that esterases produced by microorganisms are key enzymes for detoxification of pyrethroid pesticides. In this study, by performing enzyme localization studies involving the CD-9 strain, we found that the degrading enzyme produced was an intracellular enzyme, and the degradation rates of the intracellular crude enzyme solution for fenvalerate, bifenthrin, and beta-cypermethrin were 73.87%, 66.00% and 67.90%, respectively. The crude enzyme solution of strain CD-9 has high degradation efficiency for different synthetic pyrethroids (Bhatt et al. 2020e). These findings indicate the potential of the *C. freundii* CD-9 in the degradation of fenvalerate and bioremediation of pyrethroid pesticide contaminated environments, thereby contributing to degradation research involving other pesticides. Cellular enzymes are the main microbial degraders of pesticides (Zhang et al. 2019). To further develop related pesticide degradation enzyme preparations, we need to better understand the degradation mechanisms of pesticide degrading enzymes and how to select for bacteria that can degrade target pollutants and prepare mixed microbial agents.

In conclusion, a fenvalerate-degrading bacterial strain, *C. freundii* CD-9, was isolated and characterized in terms of its physiology, biochemistry, and biodegradation ability. The optimum conditions for enhanced biodegradation were determined to be initial pH 6.3, fenvalerate concentration 77 mg/L, and an inoculation amount of 6% (v/v), resulting in 88% degradation of fenvalerate within 72 h. A metabolic pathway for the degradation of fenvalerate was proposed after identifying the different intermediate compounds produced during the degradation process. Moreover, through enzyme localization studies in strain CD-9, it was found that the degrading enzyme was an intracellular enzyme. These insights will help formulate new strategies to suppress pesticide residues related to environmental pollution.

## Data Availability

The corresponding author is responsible for providing all experimental data upon request.

## Abbreviations

*GC-MS*: Gas chromatography-mass spectrometry
*HPLC*: High-performance liquid chromatography
*LB*: Luria-Bertani
*MSM*: Mineral salt medium
*RSM*: Response surface methodology
*3-PBA*: 3-phenoxy-benzoic acid
